# ICTV Virus Taxonomy Profile: Nairoviridae 2024

**DOI:** 10.1099/jgv.0.001974

**Published:** 2024-04-30

**Authors:** Jens H. Kuhn, Sergey V. Alkhovsky [Альховский Сергей Владимирови], Tatjana Avšič-Županc, Éric Bergeron, Felicity Burt, Koray Ergünay, Aura R. Garrison, Marco Marklewitz, Ali Mirazimi, Anna Papa [Άννα Παπά], Janusz T. Pawęska, Jessica R. Spengler, Gustavo Palacios

**Affiliations:** 1 Integrated Research Facility at Fort Detrick, Frederick MD, USA; 2 D.I. Ivanovsky Institute of Virology of N.F. Gamaleya National Center on Epidemiology and Microbiology of Ministry of Health of Russian Federation, Moscow, Russia; 3 University of Ljubljana, Ljubljana, Slovenia; 4 Centers for Disease Control and Prevention, Atlanta GA, USA; 5 University of the Free State, Bloemfontein, South Africa; 6 Hacettepe University Faculty of Medicine, Ankara, Turkey; 7 Smithsonian Institution, Museum Support Center, Suitland MD, USA; 8 Walter Reed Army Institute of Research, Silver Spring MD, USA; 9 Smithsonian Institution–National Museum of Natural History, Washington, DC, USA; 10 United States Army Medical Research Institute of Infectious Diseases, Fort Detrick, Frederick MD, USA; 11 FIND, Geneva, Switzerland; 12 Folkhalsomyndigheten, Stockholm, Sweden; 13 Aristotle University of Thessaloniki, Thessaloniki, Greece; 14 National Institute for Communicable Diseases of the National Health Laboratory Service, Sandringham-Johannesburg, Gauteng, South Africa; 15 Icahn School of Medicine at Mount Sinai, New York, NY, USA

**Keywords:** ICTV Report, nairovirid, *Nairoviridae*, nairovirus, orthonairovirus, taxonomy

## Abstract

*Nairoviridae* is a family for negative-sense RNA viruses with genomes of about 17.2–21.1 kb. These viruses are maintained in and/or transmitted by arthropods among birds, reptiles and mammals. Norwaviruses and orthonairoviruses can cause febrile illness in humans. Several orthonairoviruses can infect mammals, causing mild, severe and sometimes, fatal diseases. Nairovirids produce enveloped virions containing two or three single-stranded RNA segments with open reading frames that encode a nucleoprotein (N), sometimes a glycoprotein precursor (GPC), and a large (L) protein containing an RNA-directed RNA polymerase (RdRP) domain. This is a summary of the International Committee on Taxonomy of Viruses (ICTV) report on the family *Nairoviridae*, which is available at www.ictv.global/report/nairoviridae.

## Virion

**Fig. 1.**
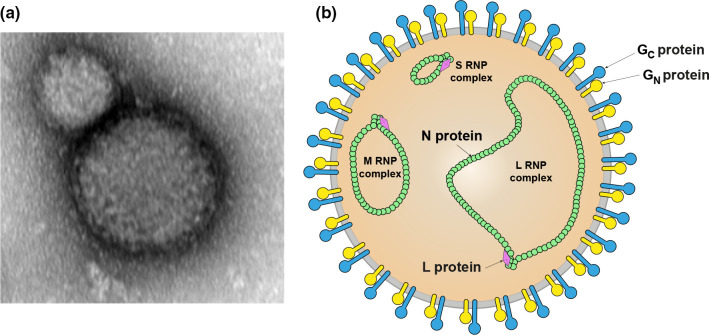
(**a**) Transmission electron micrograph of a Crimean-Congo hemorrhagic fever virus particle, magnification 50 000× (courtesy of Mateja Poljšak-Prijatelj and Marko Kolenc, Institute of Microbiology and Immunology, Ljubljana, Slovenia). (**b**) Illustration of a nairovirid particle.

Nairovirids produce virions that are spherical in shape and 80–120 nm in diameter, with lipid envelopes (Table 1 and Fig. 1). The virion surface layer of most nairovirids is covered with spikes composed of GP subunits G_N_ and G_C_. Isolated ribonucleoprotein (RNP) complexes are composed of individual segments of genomic RNA encapsidated by N protein and associated with L protein.

**Table 1.** Characteristics of members of the family *Nairoviridae*

Example	Crimean-Congo hemorrhagic fever virus (S: U88410; M: AF467768; L: AY389361), species *Orthonairovirus hemorrhagiae*, genus *Orthonairovirus*
Virion	Enveloped, spherical virions 80–120 nm in diameter with heterodimeric surface spikes
Genome	Two or three single-stranded RNA molecules (segments): small (S; 1.4–5.5 kb), medium (M; 3.3–5.9 kb), and large (L; 9.1–14.9 kb)
Replication	Ribonucleoprotein (RNP) complexes containing anti-genomic RNA serve as coding templates for synthesis of genomic RNA
Translation	From capped and non-polyadenylated mRNAs; the 5′-cap structure is obtained via cap-snatching from cellular mRNAs
Host range	Arthropods (e.g. norwaviruses, oceteviruses, orthonairoviruses, sabaviruses, shaspiviruses, striwaviruses, xinspiviruses), birds (e.g. orthonairoviruses) and mammals (e.g. orthonairoviruses)
Taxonomy	Realm *Riboviria,* kingdom *Orthornavirae*, phylum *Negarnaviricota*, subphylum *Polyploviricotina*, class *Ellioviricetes*, order *Bunyavirales*; the family includes >6 genera and >50 species.

## Genome

**Fig. 2.**
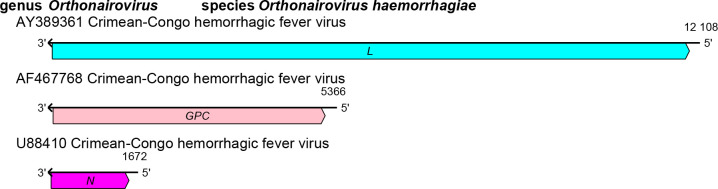
Nairovirid genome. *GPC*, glycoprotein precursor gene; *L*, large protein gene; *N*, nucleoprotein gene.

Nairovirids have bisegmented (small [S] and large [L] segments) or trisegmented (S, medium [M], and L segments) negative-sense RNA genomes (Fig. 2). These RNAs encode, in the virus-complementary sense, N (S segment), GPC (M segment), and L protein containing RdRP, helicase, and endonuclease domains (L segment).

## Replication

Nairovirid infection starts with virion attachment, mediated by G_N_ and G_C_, to unknown cell-surface receptors and entry via the endosomal route [1]. Viral fusion with the host cell results in early or late endosomal release, depending on the virus, of the virion RNP complex into the cytoplasm. This pH-dependent fusion event likely requires the previous participation of an intracellular receptor [1,2]. During primary transcription, L generates uncapped antigenomic RNAs that are then capped using host cell-derived capped primers (cap snatching) [3]. L and S segment-transcribed mRNAs are translated by free ribosomes. M segment-transcribed mRNA is translated by membrane-bound ribosomes, with the expressed GPC co-translationally and post-translationally cleaved by cellular proteases and glycosylated by cellular glycosidases to yield G_N_ and G_C_ and sometimes non-structural glycoproteins [4]. The antigenome, synthesised by L protein, serves as a template for genomic RNA replication. Secondary transcription amplifies the synthesis of mRNAs and genome replication. During morphogenesis, G_N_ and G_C_ accumulate in the Golgi apparatus, modified host membranes are acquired, and virions bud into the Golgi cisternae [5,6].

## Taxonomy

Current taxonomy: ictv.global/taxonomy. The family *Nairoviridae* is included in the negarnaviricot order *Bunyavirales*. Within this order, nairovirids are most closely related to arenavirids, discovirids, leishbuvirids, mypovirids, phenuivirids, and wupedevirids. Like most other bunyavirals, nairovirids (i) have multisegmented, negative-sense RNA genomes; (ii) encode proteins with high sequence identity; (iii) have five conserved motifs (A–E) in their RdRP domain; and (iv) produce enveloped virions.

## Resources

Full ICTV Report on the family *Nairoviridae*: www.ictv.global/report/nairoviridae.

## Abbreviations

GP: glycoprotein
GPC: glycoprotein precursor
L: large (protein/segment)
M: medium (segment)
N: nucleoprotein
RdRP: RNA-directed RNA polymerase
RNP: ribonucleoprotein
S: small (segment)
